# Rescue of tau-induced synaptic transmission pathology by paclitaxel

**DOI:** 10.3389/fncel.2014.00034

**Published:** 2014-02-10

**Authors:** Hadas Erez, Or A. Shemesh, Micha E. Spira

**Affiliations:** Department of Neurobiology, The Life Sciences Institute, The Hebrew University of JerusalemJerusalem, Israel

**Keywords:** tauopathy, mutant-human tau, paclitaxel, homosynaptic depression, vesicle stores, Aplysia

## Abstract

Behavioral and electrophysiological studies of Alzheimer’s disease (AD) and other tauopathies have revealed that the onset of cognitive decline correlates better with synaptic dysfunctions than with hallmark pathologies such as extracellular amyloid-β plaques, intracellular hyperphosphorylated tau or neuronal loss. Recent experiments have also demonstrated that anti-cancer microtubule (MT)-stabilizing drugs can rescue tau-induced behavioral decline and hallmark neuron pathologies. Nevertheless, the mechanisms underlying tau-induced synaptic dysfunction as well as those involved in the rescue of cognitive decline by MTs-stabilizing drugs remain unclear. Here we began to study these mechanisms using the glutaminergic sensory-motoneuron synapse derived from *Aplysia* ganglia, electrophysiological methods, the expression of mutant-human tau (mt-htau) either pre or postsynaptically and the antimitotic drug paclitaxel. Expression of mt-htau in the presynaptic neurons led to reduced excitatory postsynaptic potential (EPSP) amplitude generated by rested synapses within 3 days of mt-htau expression, and to deeper levels of homosynaptic depression. mt-htau-induced synaptic weakening correlated with reduced releasable presynaptic vesicle pools as revealed by the induction of asynchronous neurotransmitter release by hypertonic sucrose solution. Paclitaxel totally rescued tau-induced synaptic weakening by maintaining the availability of the presynaptic vesicle stores. Postsynaptic expression of mt-htau did not impair the above described synaptic-transmission parameters for up to 5 days. Along with earlier confocal microscope observations from our laboratory, these findings suggest that tau-induced synaptic dysfunction is the outcome of impaired axoplasmic transport and the ensuing reduction in the releasable presynaptic vesicle stores rather than the direct effects of mt-htau or paclitaxel on the synaptic release mechanisms.

## Introduction

Behavioral and electrophysiological studies of Alzheimer’s disease (AD) and other tauopathies have revealed that the onset of cognitive decline correlates better with synaptic dysfunctions than with hallmark pathologies such as the accumulation of extracellular amyloid-β plaques, intra-neuronal neurofibrillary tangles formed by aberrantly phosphorylated tau, degeneration of neurites or neuronal loss (Terry et al., 1991; Arriagada et al., 1992; Selkoe, 2002; Coleman and Yao, 2003; Giannakopoulos et al., 2003; Saul et al., 2013). Nevertheless, the mechanisms by which tau induces synaptic dysfunctions remain unclear. Studies aimed at elucidating the underlying mechanisms have drawn on the correlations between behavioral performance and synaptic structure, synaptic biochemistry and synaptic electrophysiology. Using transgenic (Tg) mice exhibiting tau-induced cognitive decline, a number of studies have indicated that loss of dendritic spine or depletion of synaptic proteins appear prior to hallmark pathologies (Eckermann et al., 2007; Yoshiyama et al., 2007; Mocanu et al., 2008; Polydoro et al., 2009; Bittner et al., 2010; Hoover et al., 2010; Rocher et al., 2010; Crimins et al., 2011; Sydow et al., 2011a,b; Alldred et al., 2012; Spires-Jones and Knafo, 2012; Kopeikina et al., 2013). Electrophysiological approaches designed to relate declined cognition and functional synaptic transmission have indicated that the expression of pathological mutant-human tau (mt-htau) leads to synaptic dysfunctions by impairing both pre and postsynaptic mechanisms (Yoshiyama et al., 2007; Polydoro et al., 2009; Hoover et al., 2010; Crimins et al., 2012; Hochgrafe et al., 2013). For example, postsynaptic impairment of long-term potentiation (LTP) at the CA1–CA3 synapses of rTgP301L mice is generated by mislocalization and accumulation of hyperphosphorylated tau in dendritic spines. This leads in turn to impaired glutamate receptor trafficking, targeting and anchoring to the postsynaptic membrane, resulting in impairment of the postsynaptic cascade associated with LTP (Hoover et al., 2010). Other postsynaptic mechanisms were also discussed by Ittner et al. (2010). A number of studies have also shown that prior to neuronal loss, htau induces synaptic dysfunctions by presynaptic mechanisms which reduce the probability of neurotransmitter release (Yoshiyama et al., 2007; Polydoro et al., 2009; Chakroborty et al., 2012; Tai et al., 2012). The presynaptic mechanisms were not investigated in details in these studies and could theoretically be the outcome of impaired axoplasmic transport or direct tau interference with the molecular machinery of neurotransmitter release (Morfini et al., 2009).

Recent behavioral studies complemented by biochemical and histological observations suggest that antimitotic drugs such as paclitaxel or epothilone D can rescue Tg tau mice from developing symptomatic behavioral decline and may even reverse it (Michaelis et al., 2002; Ballatore et al., 2007, 2012; Brunden et al., 2009, 2010a,b, 2012; Zhang et al., 2012). The rescue and putative therapeutic effects of these antimitotic drugs are attributed to their microtubule (MT) stabilizing function which counteracts MT destabilization by tau phosphorylation (Lee et al., 1994).

In a recent series of live confocal microscope studies our laboratory demonstrated that cultured *Aplysia* neurons can serve as a cell biological platform to document and analyze cellular pathologies induced by human tau and its rescue by paclitaxel (Shemesh et al., 2008; Shemesh and Spira, 2010a, 2011). We showed that the expression of wild type or mt-htau in cultured *Aplysia* neurons leads to (1) the swelling of axonal segments (Stokin et al., 2005); (2) translocation of tau to submembrane domains (Brandt et al., 1995); (3) reduction in the number of MTs along the axon; (4) the reversal of their polar orientation (Shemesh et al., 2008; Shemesh and Spira, 2010a); (5) impaired organelle transport (Stamer et al., 2002); (6) dramatic accumulation of macro-autophagosomes (Nixon et al., 2008; Shemesh and Spira, 2010b); (7) compromised neurite morphology (Kraemer et al., 2003); and (8) degeneration (Wittmann et al., 2001). In addition, using this cellular platform, we found that bath application of 10 nM paclitaxel prior to the onset of mt-htau induced pathological processes rescued the neurons from undergoing the cell pathologies described above. Higher concentrations of paclitaxel (100 nM) did not prevent the unfolding of the pathologies (Shemesh and Spira, 2011).

In the present study we used the classical glutaminergic sensory-motorneuron (SN-MN) synapse derived from *Aplysia* *californica* (Kandel, 2001) to further explore the fundamental mechanisms underlying tau-induced synaptic dysfunctions and better understand the effects of MT-stabilizing drugs in preventing it. The SN-MN synapse has proven to be extremely useful in studies of the mechanisms underlying different forms of short-, intermediate- and long-term synaptic plasticity (Kandel, 2001; Bailey and Kandel, 2008; Glanzman, 2009; Jin et al., 2011; Mayford et al., 2012). Specifically, we examined the effects of a double mutant- tau containing both missense mutations P301S and K257T (Goedert and Jakes, 2005; Shemesh et al., 2008; Shemesh and Spira, 2010a, 2011) on three well characterized parameters of the SN-MN synapse: (a) the strength of synaptic transmission as indicated by the amplitude of the excitatory postsynaptic potential (EPSP) in a rested synapse; (b) the rate and levels of homosynaptic depression; and (c) the extent to which a single bath application of 5-Hydroxytryptamine creatinine sulfate complex (5HT) induces facilitation of a depressed synapse. Estimation of the releasable vesicle pool size revealed that expression of mt-htau in the presynaptic neuron reduces the pool size, and leads to weakening of the synaptic functions. Expression of mt-htau postsynaptically for up to 5 days did not lead to alteration in the synaptic functions. Paclitaxel (10 nM) in the culture medium rescued the synapse from the pathological changes.

## Materials and methods

### Cell cultures

Sensory neurons from the pleural ganglia of adult animals (60–80 g) were cocultured with L7 or left siphon (LFS) postsynaptic motor neurons from the abdominal ganglia of juvenile (2–5 g), or adult (100 g) specimens as described by Schacher and Proshansky (1983). Briefly, animals (imported from the NIH marine resources facility at the University of Miami, Fl, USA) were anesthetized by injection of isotonic MgCl_2_ solution. The ganglia were isolated and incubated for 1.5–3 h in 0.1% protease (protease type XIV Sigma) at 34°C. The ganglia were then desheathed, and the cell bodies with their long axons were pulled out with sharp micropipettes and placed on poly-L-lysine-coated (Sigma) glass bottom culture dishes. The culture medium consisted of (in volume) 10% filtered hemolymph from *Aplysia faciata* collected along the Mediterranean coast, 40% L-15 (Sigma) supplemented for marine species and 50% artificial sea water (ASW, see below). Twenty-four hours after plating the dishes were transferred to an 18°C incubator. Intracellular microinjections of mRNA and application of paclitaxel to the bathing solution took place on the 3rd day after plating and the electrophysiological experiments were conducted 6–8 days after plating.

### Electrophysiology

Intracellular recording and stimulation by sharp glass electrodes were performed at room temperature in artificial sea water composed of NaCl 460 mM, KCl 10 mM, CaCl_2_ 10 mM, MgCl_2_ 55 mM, and HEPES [N-(2-hydroxyethyl)piperazine-N′-2-ethanesulfonic acid, Sigma] 11 mM, adjusted to pH 7.6.

The electrodes were filled with 2M KCl and had a resistance of 5–9 MΩ. Evoked EPSPs were recorded while holding the motor neuron transmembrane potential at approximately −55 mV.

Spontaneous miniature potentials were rarely recorded in culture formed by a single sensory neuron in contact with a single L7 neuron, but could be recorded using the SN-LFS synapse.

For the electrophysiological experiments the pre and postsynaptic neurons were impaled by sharp microelectrodes that were used for both current injection and voltage recordings. In each experimental session we always included control synaptic pairs in which the sensory neurons were isolated from the same adult animals as those used for the experimental groups; similarly, the L7 motoneurons were prepared from the same batch of juvenile animals. In a series of preliminary experiments conducted every day following the injection of mt-htau encoding mRNA we observed that the effect on synaptic transmission was detectable on the 3rd day of mRNA injection and therefore concentrated the documentation of the synaptic functions on the 3rd day of mRNA injection.

To estimate the size of the releasable vesicle pool we used the SN-LFS synapses and a modified protocol developed by Zhao and Klein (2002, 2004). Briefly, to maximize the availability of vesicles to be released, the synapses were exposed to 20 nM phorbol 12,13-dibutyrate (PDBu) solution for 1 min. then 50 µl of hypertonic sucrose solution (1 M) was added from a pipette onto the cells. This led within approximately 1 s to asynchronous release of synaptic potentials at high frequency for a duration of a few minutes. Since the amplitude of the unitary synaptic potentials ranged between 0.2–2.5 mV it was assumed that a fraction of the potentials reflected multi-quantal release events (Zhao and Klein, 2002). Because the release frequency in some experiments was very high the unitary events summated to a level where individual events could not be discerned. For that reason we estimated the hypertonic sucrose solution-releasable vesicle-pool-size by measuring the integral of voltage over time of the asynchronous release. Measurements were conducted for 20 s, starting 1 s after the application rather than by counting the peak of the individual events. Calculation of the integral asynchronous release was done using in-house software.

### mRNA preparation and injection

mRNAs were transcribed *in vitro* using the recombinant transcription system as described elsewhere by our laboratory (Sahly et al., 2003). Double mt-htau containing both missense mutations P301S and K257T (Shemesh et al., 2008) was cloned in a pCS2+ expression vector as previously reported . The transcribed mRNAs were pressure-injected into the cell body cytoplasm of the pre or postsynaptic cultured neurons 3 days after plating, when the synaptic contact between the cells were already established and had reached steady state strength as described earlier in Sahly et al. (2003).

### Confocal microscope imaging

The systems used for confocal imaging included a Nikon C1 system mounted on a Nikon TE-2000 Eclipse microscope with a Nikon plan-Apo chromat 60x 1.4 NA oil objective. The system was equipped with three lasers: blue diode (405 nm), argon (488 nm) and green HeNe (543 nm). Images were collected and processed using EZ-C1 software at 20–24°C as previously described (Shemesh et al., 2008; Shemesh and Spira, 2011). Cherry-tagged mt-htau was excited at 543 nm; the emitted fluorescence was collected using a 605/75 nm filter. Green fluorescent protein (GFP)-tagged mt-htau was excited at 488 nm; the emitted fluorescence was collected using a 515/30 nm filter. Cerulean tagged mt-htau was excited at 405 nm; the emitted fluorescence was collected using a 485/30 nm filter.

### Drugs

Paclitaxel (Sigma-Aldrich) and phorbol 12,13-dibutyrate (PDBu -Sigma) were stored in a stock solution of Dimethyl Sulfoxide**** (DMSO) at a concentration of 10 mM, diluted to a concentration of 10 and 20 µM in ASW (respectively) and further diluted to a working concentration of 10 and 20 nM (respectively) at the culture dish. The DMSO concentration never exceeded 0.002% in the culture dish. 5-Hydroxytryptamine creatinine sulfate complex (5HT, Sigma) was prepared fresh on the day of the experiments. A stock solution of 10 mM was diluted in double distilled water (DDW), and then diluted to a final concentration of 10 µM in ASW.

### Statistical analysis

The effects of various treatments are presented as the percent change of EPSP amplitude after treatment with respect to the initial EPSP amplitude before treatment. All the data are presented as mean ± standard error of the mean (SEM). *t*-tests and ANOVAs were performed using Excel software.

## Results

### The effects of mt-human tau expression and paclitaxel on synaptic properties

To characterize the effects of mt-htau on synaptic transmission and examine the potential use of paclitaxel to counteract it we studied four experimental groups: (a) a control group of cocultured presynaptic sensory neurons and L7 postsynaptic motor neurons. In this group the presynaptic neurons were injected on day 3 in culture with KCl or were not injected; (b) a group of synaptic pairs in which the presynaptic neurons were injected on day 3 in culture with mRNA encoding GFP-, cerulean-, or cherry-tagged double mutant-human tau containing missense mutations P301S and K257T (Shemesh et al., 2008); (c) like group *b*, but 1–2 h after tagged mt-htau mRNA microinjection, paclitaxel at a final concentration of 10 nM was added to the culture solution; and (d) the presynaptic neuron not injected with mt-htau mRNA, but rather exposed to 10 nM paclitaxel from day 3 after culturing.

All groups were subjected to electrophysiological experiments in which the resting potential, input resistances (Rin) and three synaptic parameters were monitored: (a) the amplitude of the first EPSP generated by rested synaptic pairs; (b) the time course and level of homosynaptic depression as revealed by delivery of 40 intracellular stimuli evoking presynaptic action potentials at 0.05 Hz; and (c), the extent to which a single bath application of 10 µM 5HT induced facilitation of the depressed synapse.

Confocal imaging revealed that the translational products of the injected tagged mt-htau mRNA reached detectable levels in the cytoplasm within 4 h of injection. Twenty-four hours later the fluorescently tagged mt-htau was well expressed throughout the entire sensory neuron. Using confocal imaging of the MTs and axonal transport, we documented in an earlier study (Shemesh et al., 2008) that initial impairments of the axoplasmic transport can be imaged within 24 h of the mRNA injection. With time the axoplasmic transport was further reduced.

The resting potentials of the pre and postsynaptic neurons were found to be similar in the control and experimental groups (Table 1). Whereas the averaged Rin of the presynaptic neurons expressing mt-htau and bathed in paclitaxel, and the averaged Rin in paclitaxel treated neurons are higher than the averaged input resistances of the control and mt-htau expressing neurons, the differences are statistically not significant (Table 1 row 3, *P* = 0.28 and *P* = 0.35 respectively).

**Table 1:** **Electrophysiology parameters of the pre- and postsynaptic neurons**.

	**Control**	**mt-htau**	**mt-htau & Paclitaxel**	**Paclitaxel**
Presynaptic resting	−40.87 ± 2.43	−38 ± 2.37	−42 ± 1.24	−37.5 ± 1.34
potential (mV)	*n* = 8	*n* = 6, *p* = 0.41	*n* = 9, *p* = 0.69	*n* = 8, *p* = 0.25
Postsynaptic (L7)	−53.91 ± 0.8	−56.88 ± 1.61
resting potential (mV)	*n* = 11	*n* = 16, *p* = 0.11
Presynaptic Input	54.57 ± 5.54	57.37 ± 8.21	71.69 ± 13.62	72.73 ± 17.33
resistance (MΩ)	*n* = 12	*n* = 8, *p* = 0.78	*n* = 7, *p* = 0.28	*n* = 6, *p* = 0.35
Postsynaptic (L7)	26.67 ± 4.96	19.8 ± 1.62
Input resistance (MΩ)	*n* = 7	*n* = 12, *p* = 0.29
Miniature potential	0.22 ± 0.02,	0.22 ± 0.03,
amplitude (mV)	*n* = 205, 5 cells	*n* = 119, 6 cells
Presynaptic spike	97.12 ± 1.66,	93.38 ± 2.08,	93.51 ± 3.38,	92.46 ± 6.73,
amplitude (mV)	*n* = 8	*n* = 8, *p* = 0.18	*n* = 9, *p* = 0.36	*n* = 4, *p* = 0.29
Presynaptic spike	2.59 ± 0.09	2.37 ± 0.13	2.37 ± 0.18	2.39 ± 0.2
duration (msec)	*n* = 14	*n* = 8, *p* = 0.22	*n* = 9, *p* = 0.31	*n* = 7, *p* = 0.39
Calcium peak (ΔF/F0)	0.65 ± 0.07	0.79 ± 0.04	1.17 ± 0.22	0.66 ± 0.12
Presynaptic neuron	*n* = 6	*n* = 5, *p* = 0.11	*n* = 5, *p* = 0.08	*n* = 7, *p* = 0.87

**Figure 1:**
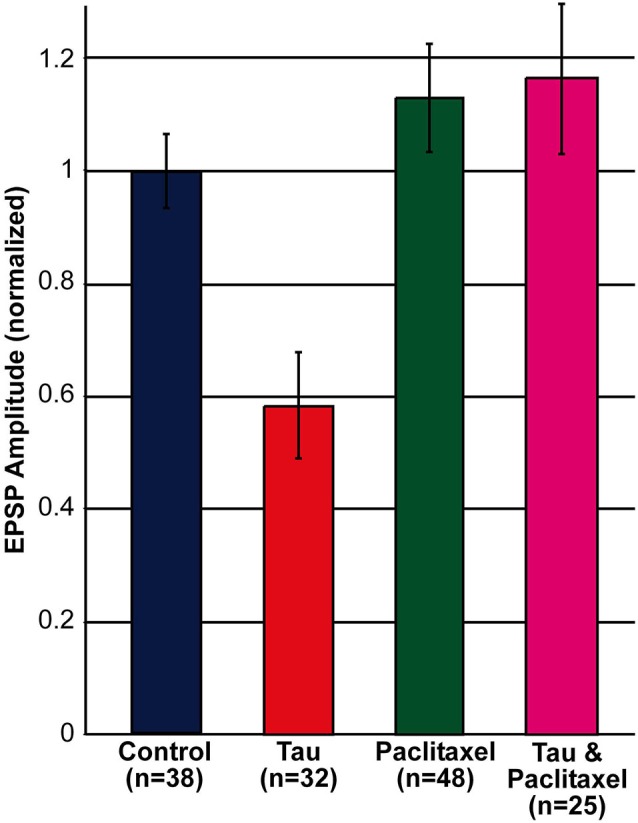
**Decreased EPSP amplitude by the expression of presynaptic mt-htau for 3 days and its rescue by 10 nM paclitaxel**. Shown are the normalized amplitudes of the first EPSP generated in rested synaptic pairs (SN-L7 and SN-LFS synapses). In blue, control—the EPSPs generated by synaptic pairs cultured for 6 days. In red, the first EPSP generated by the stimulation of a mt-htau expressing presynaptic neuron injected on day 3 in culture with fluorescently tagged mt-htau mRNA. The evoked EPSP was monitored 3 days later. In green, the first EPSP generated by synaptic pairs exposed on the 3rd day in culture to 10 nM paclitaxel for 3 days. In magenta, the EPSP generated by synaptic pairs expressing fluorescently tagged mt-htau for 3 days while being bathed in 10 nM paclitaxel since the injection of mt-htau mRNA.

Comparison of the amplitude of the first EPSP generated by stimulation of rested synaptic pairs (Figure 1) revealed that in synaptic pairs expressing presynaptic mt-htau the average first EPSP amplitude was significantly reduced by 41.62 ± 9.39% in comparison to the control EPSPs (for SN-L7 synapses from 12.82 ± 1.14 to 7.89 ± 1.65 mV, *n* = 19 and 22 for control and mt-htau respectively, *α* = 0.05, *p* = 0.0191 and for SN-LFS synapses from 22.78 ± 2.19 to 11.7 ± 2.43 mV, *n* = 19 and 10 for control and mt-htau respectively, *α* = 0.05, *p* = 0.0026. *t*-test for the normalized results of all synapses, for unequal variances, *n* = 38 and 32 for control and mt-htau respectively, *α* = 0.005, *p* = 0.00057). In neurons that were not injected with mt-htau mRNA and bathed in 10 nM paclitaxel for 3 days the average amplitude of the first EPSP was similar to that of the average control EPSPs (Figure 1) (*t*-test for normalized results of SN-L7 and SN-LFS synapses, for unequal variances, *n* = 38 and 48 for control and paclitaxel respectively, *α* = 0.05, *p* = 0.27). In synapses formed by presynaptic neurons expressing mt-htau and continuously bathed in 10 nM paclitaxel the amplitude of the first EPSP was significantly higher than in mt-htau expressing presynaptic neurons (*t*-test for normalized results of SN-L7 and SN-LFS synapses, for unequal variances, *n* = 32 and 25 for tau and tau+paclitaxel respectively, *α* = 0.005, *p* = 0.00087) but not significantly higher than the average control EPSPs (*t*-test for normalized results of SN-L7 and SN-LFS synapses, for unequal variances, *n* = 38 and 25 for control and tau+paclitaxel respectively, *α* = 0.05, *p* = 0.28).

In summary these experiments revealed that presynaptic expression of mt-htau leads within 3 days of expression to decreased amplitude of the first EPSP evoked in rested synapses, and that this effect is impeded by the continuous presence of 10 nM paclitaxel in the culture medium (from day 3 onward, Figure 1).

**Figure 2:**
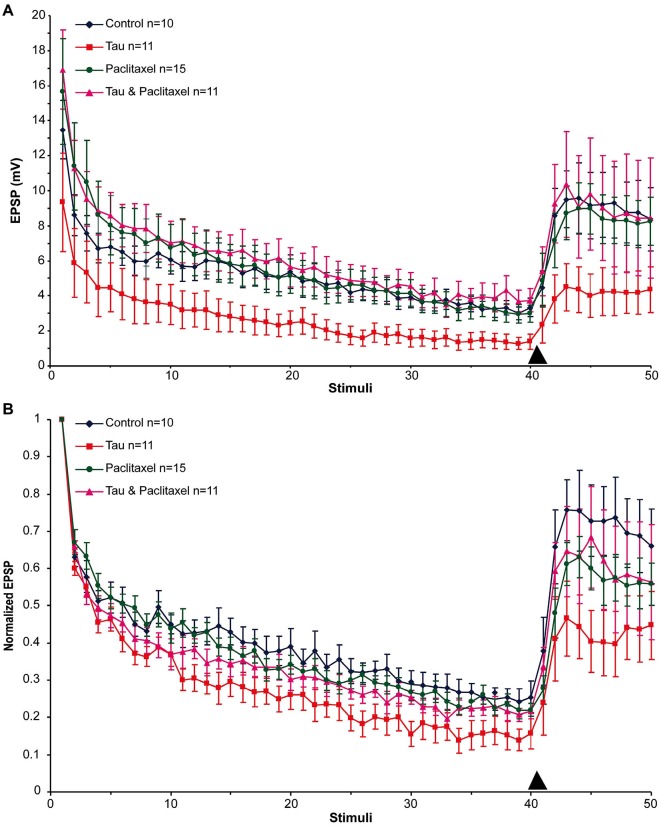
**The effects of presynaptic expression of mt-htau on homosynaptic depression kinetics, 5HT induced facilitation of the depressed synapse and the rescue of mt-htau-induced synaptic functions by paclitaxel**. All recordings were made on day 6 in culture. **(A)** Raw average data. **(B)** Normalized results. Blue-** c**ontrol neurons. Red- mt-htau expressing presynaptic neurons for 3 days (days 4–6). Green- synaptic pairs bathed from day 4 to 6 in 10 nM paclitaxel. Magenta- synaptic pairs that express presynaptic mt-htau and cultured in the presence of paclitaxel from day 4 to 6. Arrowhead- time of 5HT application.

Next we examined the effects of presynaptic mt-htau expression on: (1) the rate of homosynaptic depression (throughout the 40 stimuli); (2) the degree of synaptic depression (average of stimuli 38–40); and (3) the degree of 5HT-induced facilitation (average of stimuli 43–45). To that end, the EPSPs were depressed by 40 consecutive stimuli delivered at 0.05 Hz to the presynaptic sensory neuron in the four experimental groups. Then, 10 µM 5HT was added to the bathing solution and ten additional EPSPs were measured (Figure 2). In all groups the overall rates of the normalized homosynaptic depression curves were statistically similar (repeated-measures ANOVAs: interaction effect between control and tau—**α** = 0.05, *F*_(39,720)_ = 0.45, *p* = 0.998; interaction effect between control and paclitaxel—*α* = 0.05, *F*_(39,720)_ = 0.61, *p* = 0.971; the interaction effect between control and tau+paclitaxel—*α* = 0.05, *F*_(39,720)_ = 0.33, *p* = 0.999; and the interaction effect between paclitaxel and tau+paclitaxel—*α* = 0.05, *F*_(39,720)_ = 0.7, *p* = 0.918 and between tau and tau+paclitaxel—*α* = 0.05, *F*_(39,720)_ = 0.35, *p* = 0.999).Nevertheless,**** the final degree of the normalized synaptic depression value was affected by mt-htau and ameliorated by paclitaxel (Figure 2B). In the control experiments, 40 stimuli depressed the EPSP to 24.76 ± 3% of the first EPSP (*n* = 10). In tau expressing presynaptic neurons the EPSP was depressed to 14.96 ± 3.38% of the first EPSP (significantly lower than the control, *t*-test for unequal variances, *n* = 10 and 11 for control and tau respectively, *α* = 0.05, *p* = 0.043, Figure 2). In the mt-htau expressing presynaptic neuron cultured in the presence of paclitaxel the value of the depressed EPSP was similar to the control, 21.23 ± 1.7% of the initial level (*t*-test for unequal variances, *n* = 10 and 11 for control and tau+paclitaxel respectively, *α* = 0.05, *p* = 0.32). In paclitaxel alone the depressed EPSP value did not significantly differ from the control, at 22.62 ± 2.03% of the initial level (*t*-test for unequal variances, *n* = 10 and 14 for control and paclitaxel respectively, *α* = 0.05, *p* = 0.56).

Following homosynaptic depression of the control synapses, 5HT application led to the recovery of the EPSP amplitude to 74.65 ± 9.45% of the initial level (*n* = 9) (Figure 2B). In the mt-htau expressing presynaptic neurons 5HT application led to recovery of the depressed EPSP amplitude to a lower average level of 43.75 ± 8.83% with respect to the initial amplitude (this value was significantly different from the control, *t*-test for unequal variances, *n* = 9 and 11 for control and tau respectively, *α* = 0.05, *p* = 0.028). In synapses formed between presynaptic neurons expressing mt-htau that were continuously bathed in 10 nM paclitaxel, the 5HT application induced EPSP facilitation to 65.9 ± 11.5% of the initial level (*t*-test for unequal variances, *n* = 11 and 8 for tau and tau+paclitaxel respectively, *α* = 0.05, *p* = 0.15; *t*-test for equal variances, *n* = 9 for control and 8 for tau+paclitaxel respectively, *α* = 0.05, *p* = 0.56). Finally, in synapses bathed in 10 nM paclitaxel for 3 days 5HT induced synaptic facilitation to a value closer to the control 61.38 ± 5.26% of the initial level (*t*-test for unequal variances, *n* = 9 and 15 for control and paclitaxel respectively, *α* = 0.05, *p* = 0.24).

In conclusion, presynaptic expression of mt-htau reduced the EPSP amplitude generated by stimulation of a rested synapse (Figures 1, 2), led to a greater level of homosynaptic depression and reduced 5HT-induced synaptic dishabituation (Figure 2). 10 nM paclitaxel in the culture medium rescued all these forms of mt-tau-induced syatnaptic weakening.

### Mechanisms underlying mutant-human tau-induced synaptic pathology

Recent studies have suggested that tauopathies may spread across synapses from one neuron to another (Frost and Diamond, 2010; Wu et al., 2013). Therefore, in examining possible mechanisms to account for the synaptic weakening by presynaptic expression of mt-tau we considered both pre and postsynaptic mechanisms. Theoretically the postsynaptic mechanisms could include reduced resting potential of the motoneuron, reduced input resistance, reduced receptor density and/or receptor conductance. The presynaptic mechanisms could include decreased transient elevation of the free intracellular calcium concentration following the firing of a presynaptic action potential, reduced amounts of readily releasable vesicle store, or impairments of the release machinery.

Since the resting potential, input resistance of L7 and the amplitude of the miniature potentials recorded from LFS neurons were not significantly altered in synapses expressing presynaptic mt-htau (Table 1) it is reasonable to assume that within the time frame of the experiments, presynaptic expressing of mt-htau did not induce anterograde trans synaptic alterations.

Therefore, we examined whether presynaptic expression of mt-htau reduces evoked release by impairing the mechanism of the spike generated voltage dependent calcium influx. To that end, we recorded the presynaptic action potential by intracellular microelectrodes inserted into the cell body and the associated transient elevation of the free intracellular calcium concentrations by confocal microscope imaging of fluo-4 from the axon of isolated sensory neurons or sensory neurons forming synaptic contact with L7. These observations revealed that mt-htau expression did not interfere with the spike generation mechanisms and the ensuing transient elevation of the bulk free intracellular calcium levels in presynaptic neuron (Table 1).

**Figure 3:**
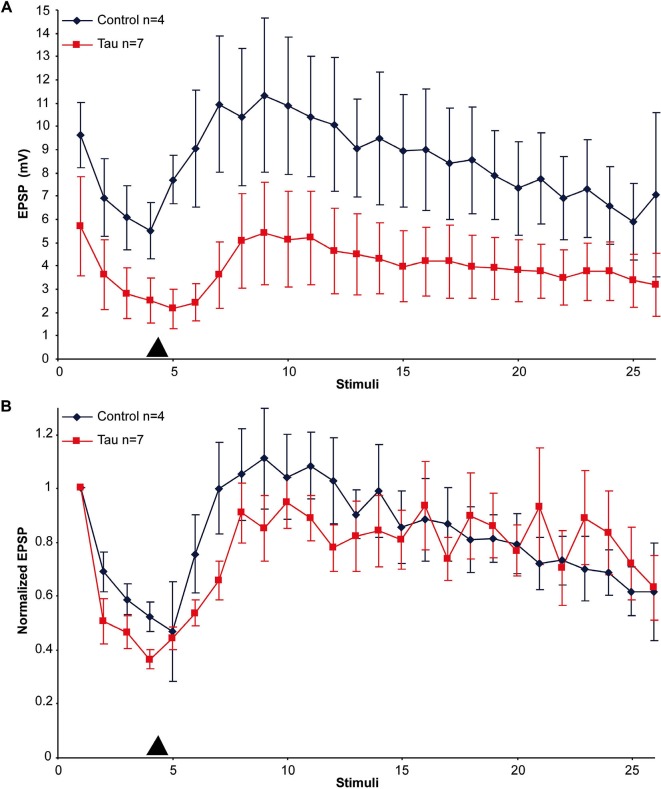
**PDBu induced facilitation of moderately depressed control- and mt-htau expressing synapses**. **(A)** Raw data. **(B)** Normalized results. For the experiments presynaptic neurons expressing mt-htau for 3 days (from day 4 to 6) were stimulated four times at a rate of 0.05 HZ. Thereafter PDBu was bath-applied to a final concentration of 20 nM. Note that although the average absolute amplitude of the EPSP in the mt-htau expressing synapses is smaller, the rates of synaptic depression and 5HT-induced facilitation were similar in the control (blue) and the presynaptically expressing mt-htau neurons (red). Arrowhead- time of application of 20 nM PDBu.

An alternative hypothesis to account for the impaired synaptic transmission is that mt-htau expression interferes with the mechanisms of vesicle translocation from the reserve store to the readily releasable pool and/or from the cell body to the synaptic terminals. Earlier studies showed that in cultured *Aplysia* SN-L7 neurons, PDBu application leads to vesicle mobilization from a reserve pool to a readily releasable one (Gingrich and Byrne, 1985; Hochner et al., 1986; Bailey and Chen, 1988; Braha et al., 1990; Dale and Kandel, 1990; Ghirardi et al., 1992; Manseau et al., 2001; Khoutorsky and Spira, 2005). To examine the first hypothesis we compared the time course and maximal amplitude of PDBu induced EPSP facilitation in moderately depressed synapses of control and mt-htau expressing neurons (Figure 3). In the experiments we applied four presynaptic stimuli that partially depressed the control synapses to 55.26 ± 5.39% and the presynaptically expressing mt-htau neurons to 40.26 ± 3.44% of the first EPSP. Then 20 nM PDBu was applied to the bathing solution. This led to increased EPSPs amplitudes in both the control and the experimental synaptic pairs with no significant difference between the two (*t*-test for unequal variances, *n* = 4 and 7 for control and mt-htau respectively, *α* = 0.05, *p* = 0.24, average of stimulus 7–9). In both, the maximal EPSP amplitude was reached after five stimuli (100 s). These observations suggested that vesicle mobilization activated by PDBu was not impaired by mt-htau expression.

### Estimation of the changes in the vesicle pool size induced by mutant-human tau expression and paclitaxel

The observations described above could reflect partial depletion of vesicle stores (Bykhovskaia, 2011) at the presynaptic terminal due to mt-htau-induced impairment of the axoplasmic transport (Shemesh and Spira, 2011). To examine this hypothesis and test whether paclitaxel prevents such reduction from occurring, we estimated the amount of transmitter stores in the presynaptic terminals by bath application of hypertonic sucrose solution. In earlier publications Zhao and Klein (2002, 2004) showed that this technique can be used to assess the vesicle pool size in cultured *Aplysia* neurons, as in other synapses (Rosenmund and Stevens, 1996; Zhao and Klein, 2002, 2004).

**Figure 4:**
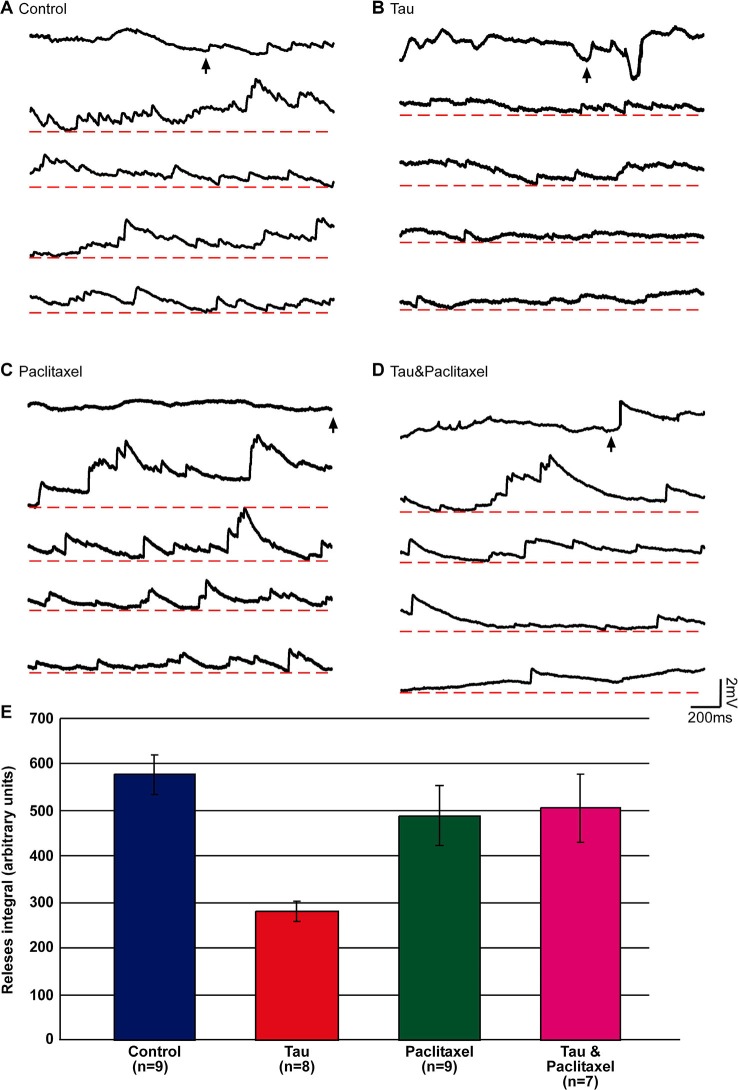
**Estimation of the releasable presynaptic vesicle pool size in control, mt-htau expressing presynaptic neurons, and the effects of paclitaxel**. The releasable presynaptic vesicle pool size of four experimental groups was estimated by monitoring asynchronous postsynaptic potentials from SN-LFS synapses following bath application of hypertonic sucrose solution. **(A–D)** are sample recordings of the sucrose-evoked asynchronous potentials. **(E)** The integral of the amplitude of asynchronous release over time. **(A)** Control, neurons grown for 6 days in culture (blue in **E**). **(B)** The presynaptic neurons were injected on day 3 in culture with mt-htau mRNA and tested on day 6 by application of hyper osmotic sucrose solution (red in **E**). **(C)** Neurons exposed to paclitaxel for 3 days (from day 3 to 6) and tested on day 6 by hyperosmotic sucrose solution (green in **E**). **(D)** Like **B**, but also exposed to paclitaxel from day 3 (magenta in **E**). The arrows in **A**, **B**, **C** and **D** depict the time of sucrose application. **(E)** The integral of voltage over time of the asynchronous release sampled five times over a period of 2 s (10 s), starting 1 s after the application.

In the experiments, presynaptic SNs and postsynaptic LFS neurons were cocultured for 3 days and the four experimental groups and protocols described above were tested. In each experiment we first applied a single stimulus to the presynaptic neuron to test whether a functional synapse was indeed established. To increase the probability of neurotransmitter release, we next applied 20 nM PDBu to the bathing solution. One minute later, 50 µl of 1 M Sucrose solution was applied to the culture dish (containing 3 ml ASW). Bath application of 50 µl, 1 M sucrose led within less than a second to asynchronous release of neurotransmitter that appeared as a barrage of synaptic potentials representing single vesicles (miniature potentials) as well as multi-quantal release events (Figure 4). The amplitudes of the asynchronous potentials ranged in different experiments from 0.2 to 2.5 mV. The frequency of release (as estimated by counting the peaks of the potentials) was in the range of 0.4–16.5 Hz. It should be noted, however, that at high frequencies the asynchronized potentials summated (Figure 4A). Therefore, we estimated the size of the hyperosmotic sucrose-releasable neurotransmitter pool by measuring the voltage integral of the asynchronized potential amplitudes over time rather than by counting the peaks of individual events (Figure 4B; Zhao and Klein, 2002, 2004).

Using this approach we found that the hypertonic sucrose solution-releasable vesicle pool size was reduced to 48% in presynaptic neurons expressing mt-htau (estimated by the total number of releases, see Materials and Methods; *t*-test for unequal variances, *n* = 9 and 8 for control and mt-htau respectively, *α* = 0.05, *p* = 0.00005). Continuous bathing of the mt-htau expressing synapses in 10 nM paclitaxel (from day 3 onward) prevented this from occurring (*t*-test for unequal variances, *n* = 9 and 7 for control and tau+paclitaxel respectively, *α* = 0.05, *p* = 0.41). No effects on the sucrose releasable pool size was observed in synapses incubated in paclitaxel alone (*t*-test for equal variances, *n* = 9 for control and paclitaxel respectively, *α* = 0.05, *p* = 0.27) (Figure 4). These observations are consistent with earlier reports from our laboratory showing that the expression of mt-htau in cultured *Aplysia* neurons leads to impaired axoplasmic transport and that paclitaxel rescues this from occurring (Shemesh et al., 2008; Shemesh and Spira, 2010a,b, 2011).

### Postsynaptic effects of mutant-human tau (mt-htau) expression

**Figure 5:**
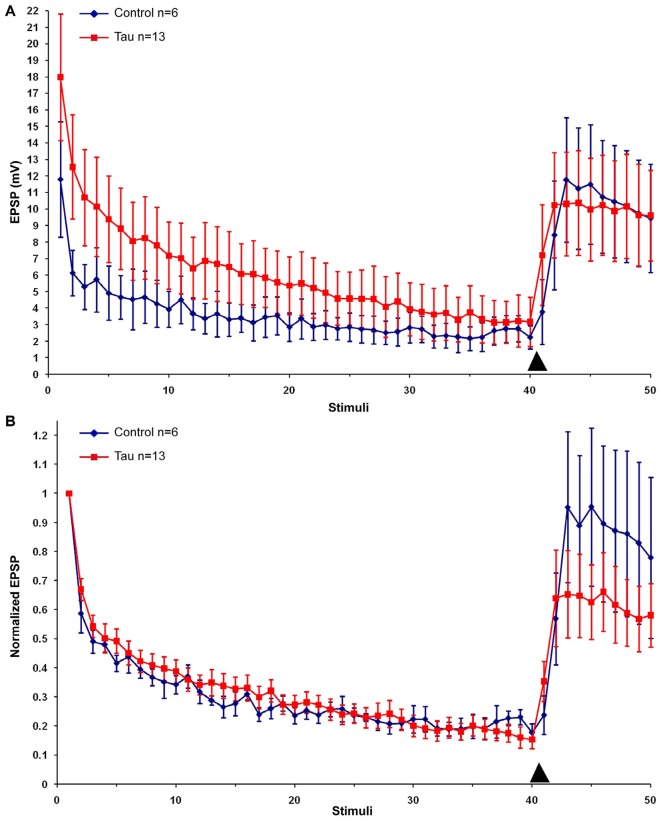
**The effects of postsynaptic expression of mt-htau on homosynaptic depression kinetics and 5HT induced facilitation of the depressed synapse**. All recordings were made on day 6 in culture. **(A)** Raw data. **(B)** Normalized results. Control neurons (blue), red- mt-human tau expressing postsynaptic neurons for 3 days from day 4 to 6 in culture. Arrowheads indicate the time of 5HT application.

Using identical protocols to those described above for mt-htau expression by the presynaptic neurons we next examined the consequences of mt-htau expression by the postsynaptic neurons L7 on synaptic transmission. Examination of the L7 trans- membrane potential and input resistance (Table 1) did not reveal any significant effects. Likewise, the EPSP amplitude of a rested control synapse and postsynaptic mt-htau expressing synapse were not significantly different (11.53 ± 2.96 and 17.99 ± 3.82 mV, *n* = 7 and 13 respectively. *t*-test for unequal variances, *α* = 0.05, *p* = 0.2). The overall rates of the normalized homosynaptic depression of control synapses and postsynaptic expressing mt-htau were statistically similar (repeated-measures ANOVAs: interaction effect between control and postsynaptic tau—*α* = 0.05, *F*_(39,400)_ = 0.32, *p* = 0.9999).**** The degree of synaptic depression (average of stimuli 38–40) were similar in the control experiments (21.23 ± 2.54% of the first EPSP) and the tau expressing postsynaptic neurons (16.38 ± 3.2%; *t*-test for unequal variances, *n* = 6 and 13 for control and postsynaptic tau respectively, *α* = 0.05, *p* = 0.25, Figure 5). 5HT-induced heterosynaptic facilitation 3 and 5 days after the injection of mt-htau mRNA to L7 did not reveal any significant changes from control (Figure 5). In the control the EPSP amplitude recovered to to 93.24 ± 25.56% of the initial level (*n* = 6) and in the mt-htau expressing postsynaptic neurons 5HT application led to recovery of the depressed EPSP amplitude to an average level of 64.29 ± 13.94% (this value were not significantly different, *t*-test for unequal variances, *n* = 6 and 12 for control and postsynaptic tau respectively, *α* = 0.05, *p* = 0.35).

In conclusion, postsynaptic expression of mt-htau does not affect the presynaptic release properties and short term homosynaptic depression which reflect presynaptic mechanisms. Possible mechanisms to explain the lack of postsynaptic mt-htau expression on synaptic transmission are discussed below.

## Discussion

The main finding of the present study is that within 3 days of mt-htau expression in the presynaptic neuron the releasable presynaptic vesicle pool is significantly reduced, resulting in diminished synaptic transmission. Continuous exposure of the neurons to 10 nM paclitaxel (from day 3 in culture onward), a clinically approved anti-neoplastic agent (Jordan and Kamath, 2007), counteracts the development of mt-htau-induced synaptic transmission dysfunctions. Along with earlier findings from our laboratory (Shemesh et al., 2008; Shemesh and Spira, 2010a,b, 2011), the present study suggests that tau-induced impaired axoplasmic transport may be the cornerstone of the cascade underlying presynaptic dysfunctions and that protection of MT by antimitotic drugs from undergoing mt-htau induced pathology is sufficient to rescue the neuron from synaptic weakening.

The present study utilized the technical advantages of the *Aplysia* cell biological platform to investigate the electrophysiology of mt-htau induced synaptic pathology. These include the ability to express mt-htau either in the pre or postsynaptic neuron, to intracellularly record and stimulate the pre or postsynaptic neurons, the ability to express mt-htau at any selected point in time and to live-image it for an extended period of time. Earlier studies from our laboratory showed that the cell biological pathologies induced by mt-htau in cultured *Aplysia* neurons are similar to those documented in vertebrates and include reduction in the number of MTs along the axon, reversal of the MT polar orientation, impaired organelle transport, accumulation of macro-autophagosomes and lysosomes (Shemesh et al., 2008; Shemesh and Spira, 2010a,b, 2011). The fundamental electrophysiological results described here demonstrate for the first time that paclitaxel rescues cultured neurons from undergoing mt-htau-induced synaptic transmission pathology.

Below we discuss possible mechanisms by which mt-human tau impairs synaptic transmission when expressed presynaptically, how 10 nM paclitaxel counteract tau induced presynaptic pathophysiology and why postsynaptic expression of mt-htau has no significant effect on the system as used.

### Mechanisms underlying synaptic dysfunction by mutant-human tau (mt-htau) expression

Impairment of axoplasmic transport mechanisms is considered a central common denominator in a variety of neurodegenerative diseases (Morfini et al., 2009; Riemer and Kins, 2013). It is reasonable to assume that even minor interferences with the steady-state maintenance of the presynaptic vesicle stores, mitochondria, other subcellular organelles, and mRNA particles may progressively lead to synaptic and neuronal circuit dysfunction which will eventually culminate in declined cognition.

The observations described above suggest that within the time frame of the experiments, mt-htau partially impairs synaptic functions by interfering with the normal steady-state maintenance of presynaptic vesicle stores. Expression of mt-htau in the postsynaptic neuron for 5 days did not lead to detectable synaptic dysfunctions.

The fact that expression of mt-htau in the presynaptic neuron does not totally block evoked release, and spike-evoked transient elevation of the intracellular calcium concentration while impairing synaptic strength and plasticity may be related to a gradual cumulative effect of impaired vesicle transport mechanisms due to the documented effects of mt-htau on the MT density and polar orientation (Shemesh et al., 2008; Shemesh and Spira, 2010a). This conclusion is consistent with earlier biochemical studies of vertebrate neurons showing that tau-hyperphosphorylation and misfolding impair axoplasmic transport lead to depletion of essential molecular components at distal neuronal sites (Hall et al., 2001; Stamer et al., 2002; Coleman and Yao, 2003; Mandelkow et al., 2003; Baas and Qiang, 2005; Hollenbeck and Saxton, 2005; Thies and Mandelkow, 2007; Cuchillo-Ibanez et al., 2008; Dixit et al., 2008; Morfini et al., 2009; Stoothoff et al., 2009; Zempel et al., 2010).

Although tau compromised the absolute amplitudes of EPSPs and led to a significantly deeper homosynaptic depression, the kinetics of 5HT or PDBu induced synaptic facilitation were not altered. Therefore it is reasonable to assume that tau does not have a direct effect on vesicle mobilization from the reserve stores to the readily releasable pool within the presynaptic terminal. Rather, by interfering with the organelle transport system along the axons, the steady state processes of maintaining the presynaptic vesicle pool is disrupted. This interpretation is consistent with the protective effects of paclitaxel on synaptic function, since paclitaxel replaces the phosphorylated tau that dissociates from neuronal MTs (Lee et al., 1994) and thereby maintains their structural and functional integrity.

In rodent CNS neurons a significant fraction of the presynaptic releasable vesicle stores is replenished by local membrane recycling (Sudhof, 2004; Schweizer and Ryan, 2006) and thus the vesicle store is partially independent of the anterograde supply of vesicles. Thus, impairment of presynaptic anterograde vesicle transport is not expected to lead to immediate depletion of the releasable neurotransmitter store even if high frequency stimuli are applied for as long as effective membrane cycling mechanisms are operative. To the best of our knowledge, in *Aplysia* SN-MN membrane cycling at the presynaptic terminal following potassium depolarization has only been documented in a single paper (Fioravante et al., 2007). Nevertheless, it is not clear to what extent local membrane cycling mechanism is effective under conditions of electrical stimulation. Therefore, it is conceivable that depletion of the presynaptic releasable vesicle pool (by impaired axoplasmic transport) has a significant effect on synaptic transmission.

The observation that presynaptic mt-htau expression leads to reduced synaptic transmission but does not impair the release processes is consistent with studies conducted on htau Tg mice and drosophila. Based on paired pulse facilitation experiments and high frequency stimulation to induce LTP, Polydoro et al. (2009) reported that the Schaffer collateral-CA1 pyramidal cells synapses of htau Tg mice reveal a decreased probability of neurotransmitter release and lack of LTP. The documented decreased release probability could be due to a number of mechanisms, one of which is a reduction in the availability of releasable vesicles as described here. Electrophysiological experiments conducted on neuromuscular junctions of Tg drosophila expressing human tau—0N3R (Chee et al., 2005, 2006) revealed that low frequency stimulation (1 Hz) of the wild type and Tg fly generate EPSPs of similar amplitudes. Nevertheless, whereas the EPSP amplitude generated at high frequency (50 Hz) was sustained by the wild-type, whereas in the Tg drosophila the EPSP amplitude underwent significant reduction. Chee et al. (2005, 2006) attributed the diminished release at high frequencies to impaired axoplasmic transport of mitochondria to the presynaptic terminals of the motor neurons, but this could also be due to depletion of vesicle stores.

Another mechanism that should be considered to account for reduction in the availability of releasable vesicle store is clustering of vesicles remote from the release sites by mt-htau. It is well established that un-phosphorylated synapsin tether synaptic vesicles to the cytoskeleton and thereby regulate the availability of neurotransmitter for release (Llinas et al., 1985). Intracellular microinjection of human tau42 recombinant protein into the presynaptic terminal of the squid giant synapse significantly reduced neurotransmitter release when stimulated at high frequencies 30–40 min after its intracellular microinjection (Moreno et al., 2011). It was suggested that the injected recombinant human-tau42 protein led to vesicle clustering away from the active zone concomitant with the reduced vesicle count at the active zone (reduced readily releasable pool). We cannot rule out that such a mechanism also contributed to the reduced synaptic functions in our experiments. Since paclitaxel competes with tau on its MT binding site it is theoretically possible that it might interfere with vesicle clustering as well.

### Why are there no significant postsynaptic mutant-human tau (mt-htau) effects on the sensory-motoneurons (SN-MN) synapse?

Electrophysiological studies conducted on Tg mouse models have attributed tau-induced synaptic anomalies to postsynaptic mechanisms generated by mislocalization and accumulation of hyperphosphorylated tau to the dendritic spines, the impairment of glutamate receptor trafficking, targeting and anchoring to the postsynaptic membrane (Hoover et al., 2010; Ittner et al., 2010; Ittner and Gotz, 2011; Kremer et al., 2011; Pozueta et al., 2013; Tai et al., 2012; Yu and Lu, 2012; Yu et al., 2012). These processes depend on the transport of AMPAR containing vesicles into dendritic spines and the fusion of the vesicles with the postsynaptic membrane. Increased tau levels in Tg mice lead to its accumulation together with protein kinase FYN in dendrites where it phosphorylates NMDA receptors and facilitates its interaction with the postsynaptic density protein PSD95.

The experimental paradigms used in the present study revealed the effects of mt-htau and paclitaxel on presynaptic mechanisms. The observation that postsynaptic expression of mt-htau for up to 5 days does not affect synaptic transmission may be taken to imply that: unlike in vertebrate neurons postsynaptic expression of mt-htau is not effecting postsynaptic mechanisms or that mt-htau is not transported retrogradely across the synaptic cleft in large enough quantities to reduce the presynaptic vesicle stores. It should be noted however that in the *Aplysia* SN-MN synapse short term plasticity in the form of homosynaptic depression or short-term facilitation involve presynaptic mechanisms. In contrast, intermediate- and long-term synaptic facilitation of the SN-MN synapse involve postsynaptic mechanisms including modulation of AMPA receptor trafficking and local protein synthesis (Roberts and Glanzman, 2003). It is thus conceivable that expression of mt-htau in the postsynaptic motor neuron would generate postsynaptic pathologies on intermediate- and long-term postsynaptic processes. This possibility was not examined in the present study and will be the subject of future studies.

### Implications of the results for clinical applications of antimitotic drugs in preventing the progress of tauopathies

Behavioral studies on rodent models and our earlier cell biological studies supported the initial hypothesis of Lee et al. (1994) that MT stabilizing reagent provide protection in both tau- and A β-induced neurodegeneration (Michaelis et al., 1998, 2005; Brunden et al., 2010b, 2012; Shemesh and Spira, 2010a,b, 2011; Ballatore et al., 2012; Zhang et al., 2012). Because paclitaxel does not permeate the blood brain barrier, recent efforts have been directed toward examining the potential use of BBB-permeable MT stabilizing reagents. These studies demonstrated that the MT-stabilizing drug Epotilone D rescue cognitive decline of Tg mice (Michaelis et al., 2002; Brunden et al., 2009, 2010a,b, 2012; Ballatore et al., 2012; Zhang et al., 2012). When considering the use of a MT stabilizing reagent to slow down, protect or even reverse tau-pathologies it is important to recall that over stabilization of dynamic MTs have direct pathological effects on neurons and other cell types and that the range of safe concentrations of these drugs may be narrow. In addition, assuming that a safe concentration can be controlled under *in vivo* conditions, the long-term use of these reagents may create secondary problems. For example, stabilized MTs by exogenous drugs may undergo acetylation or detyrosination and as a consequence be accessible to cleavage by endogenous katanin (Peris et al., 2009; Sudo and Baas, 2010). This may lead to impaired transports and would require further molecular interventions to control such secondary damage. Therefore when considering the use of MT stabilizing reagents for clinical applications these effects should be taken into consideration.

## Conflict of interest statement

The authors declare that the research was conducted in the absence of any commercial or financial relationships that could be construed as a potential conflict of interest.
